# Clinical case seminar: unraveling the mystery of abnormal thyroid function tests

**DOI:** 10.1186/s40842-015-0010-8

**Published:** 2015-09-11

**Authors:** Ariel Barkan, Ronald J. Koenig

**Affiliations:** 1 Division of Metabolism, Endocrinology and Diabetes, University of Michigan, Ann Arbor, MI USA; 2 Department of Neurosurgery, University of Michigan, Ann Arbor, MI USA; 3 24 Frank Lloyd Wright Drive, G-1500, Ann Arbor, MI 48106 USA

## Abstract

A 53 year old woman was referred to us because of large goiter, enlarged pituitary and grossly elevated TSH and free T4. The differential diagnosis included a TSH producing adenoma vs. artifactual laboratory tests. A careful step-by step analysis of different possibilities allowed correct diagnosis and treatment.

*In this Clinical Case Seminar, a real patient’s information (in bold font) and an expert clinician’s comments (in regular font) are presented for the reader. The authors’ comments are in the Background and Summary.*

## Background

The constellation of a goiter, enlarged pituitary and grossly elevated TSH and free T4 suggest a TSH producing adenoma. However, when the patient also has high titers of thyroglobulin and thyroid peroxidase antibodies, artifactual laboratory tests should also be considered in the differential diagnosis.

## Case presentation

**Fig. 1:**
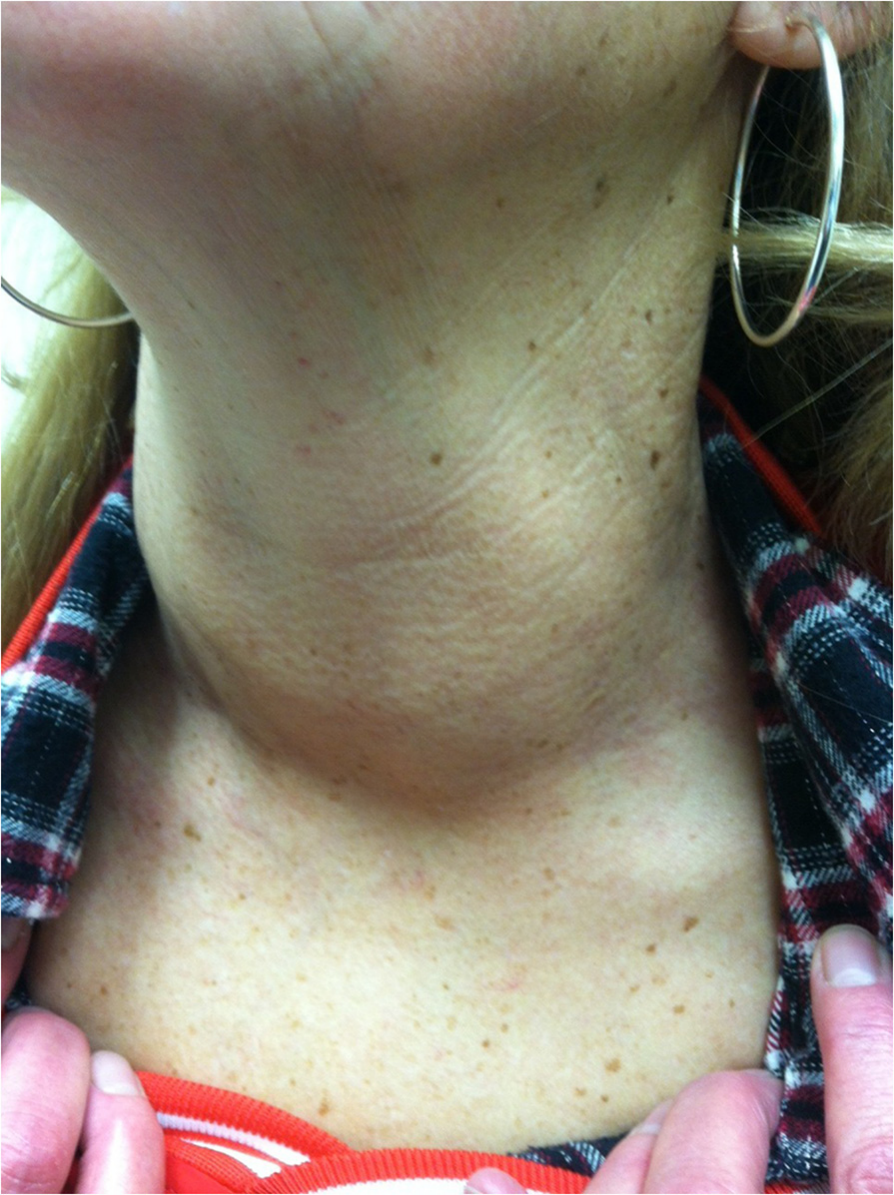
Photograph of the patient’s neck with large goiter easily visible

**The patient is a 53 year old woman who was evaluated for a large goiter but was otherwise well. Laboratory evaluation revealed a markedly elevated TSH of 82 (Normal: 0.55-4.78 mIU/L) and extremely elevated levels of thyroglobulin antibodies (246,617; Normal: 0–40 IU/mL) and thyroid peroxidase antibodies (47,097; Normal: 0–35 IU/mL) She recalled having a goiter since at least her teenage years, indicating the condition is long standing, perhaps even congenital or genetic. Over the past several years the size of the goiter had increased to a degree that was cosmetically troublesome and did not allow her to button her collars (Fig.** 1 **). On the other hand, growth and development were normal and she presented in a clinically euthyroid state, implying that the clinical consequences of the disorder have been minimal. The family history revealed hypothyroidisim in the patient’s mother, but was otherwise unremarkable.**

The constellation of goiter, extremely high TSH and grossly elevated thyroid antibodies strongly suggest the diagnosis of Hashimoto’s thyroiditis with primary hypothyroidism. Surprisingly, she had no symptoms or signs of clinical hypothyroidism.

**Unexpectedly, she was found to have markedly elevated level of Free T4 at 5.5 (Normal: 0.8-1.8 ng/dL), and a laboratory error was excluded by repeat values of TSH 125 mIU/L and FT4 5.2 ng/dL.**

**Fig. 2:**
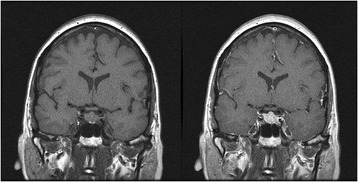
Pituitary MRI study. *Left*: T1 without contrast, *Right*: T1 with gadolinium contrast. Pituitary gland is symmetrically enlarged and enhances homogenously with gadolinium contrast

**The referring physician considered a TSH-secreting pituitary tumor or thyroid hormone resistance as potential diagnoses, and ordered additional tests. This further workup revealed mildly elevated alpha subunit of pituitary glycoprotein hormones (2.1; Normal: 0–1.4 ng/mL), and low Free T3 (2.0; Normal: 2.3-4.2 pg/mL). Thyroid ultrasound demonstrated diffusely-enlarged gland without any nodules and I-123 imaging revealed an enlarged thyroid that lacked dominant masses or distinct hot or cold nodules. Pituitary MRI revealed a diffusely enlarged gland with a minor degree of suprasellar expansion but no aggressive features. The gland was isointense on T1 without contrast and diffusely and homogenously hyperintense on T1 with gadolinium contrast (Fig.** 2 **).**

**The patient was referred to our clinic.**

**On physical examination she appeared to be well and had no symptoms or signs of hypothyroidism or thyrotoxicosis. She had a large, easily visible goiter of at least 80 ml in volume. Neck circumference was 15 inches. Other than that she had no pathological findings. She was not taking any medications with the exception of occasional NSAIDs or multivitamins.**

The differential diagnosis of an elevated TSH with an elevated FT4 in the presence of an enlarged pituitary and diffuse goiter is focused on either a pituitary TSH-producing tumor or on thyroid hormone resistance.

The lack of any signs or symptoms of hyperthyroidism despite the FT4 of 5.2 ng/dl argues against a TSH-secreting tumor, since in that case we would expect the markedly elevated FT4 to have clinical consequences. Furthermore, the FT3 would be expected to be elevated roughly in proportion to the FT4, yet in this patient the FT3 was low. The pituitary alpha glycoprotein subunit was elevated, as would be expected with a TSH-secreting adenoma. However, this 53 year old woman was post-menopausal and the elevated alpha subunit could easily be accounted for by the appropriately-elevated LH and FSH. Diffuse pituitary enlargement with characteristic pattern of contrast enhancement could simply reflect pituitary hyperplasia due to the trophic effects of longstanding TRH stimulation, consistent with her long history of goiter and presumed longstanding elevated TSH [1].

Similarly, the diagnosis of thyroid hormone resistance is not fully consistent with this case. Thyroid hormone resistance is a congenital condition, often but not always inherited in an autosomal dominant pattern [2]. Most cases are due to mutations in thyroid hormone receptor beta that decrease the affinity of the receptor for T3. The history of goiter at a young age is consistent with this diagnosis, as is the more-or-less clinically euthyroid state and the presence of elevated TSH and FT4. However, in thyroid hormone resistance, the TSH is only mildly elevated or sometimes in the upper half of the reference range, which still is inappropriate in the context of an elevated FT4. Another major difficulty invoking the diagnosis of thyroid hormone resistance is the fact that this patient has a low FT3. In thyroid hormone resistance, the FT3 should be elevated roughly in proportion to the FT4 (similar to TSH-secreting adenoma).

In fact, the most striking aspect of the laboratory evaluation is the discordance between the FT4 (elevated) and FT3 (low). Since T4 is the precursor to T3, it is difficult to square these findings. One might postulate that the low FT3 is in fact the fundamental problem, leading to the elevated TSH, which then leads to the elevated FT4 in an attempt to compensate. The clinically euthyroid state might be consistent with the only slightly low FT3 as well as perhaps contributions of the elevated FT4 to thyroid hormone action, either by direct binding of T4 to T3 receptors or by local conversion to T3 by the type 2 deiodinase. But how could the T3 be selectively low? Perhaps there is excessive activity of a T3-degrading enzyme. The problem with this hypothesis is that T3 and T4 are degraded by the same enzyme (type 3 deiodinase) [3]. One might postulate a mutant type 3 deiodinase that avidly degrades T3 in preference to T4, but there is no precedent for any such change in enzyme specificity.

How about, then, a defect in the conversion of T4 to T3? Amiodarone can block T4 to T3 conversion. Amiodarone administration results in a transient elevation of TSH, but the level rarely exceeds 15 mIU/L and is expected to return to the reference range within a few months of initiation of therapy [4]. Amiodarone causes a chronic elevation in FT4 without an elevation in FT3, but the FT4 is only slightly elevated. And of course this patient was not taking amiodarone. Still, the response to amiodarone establishes a principle. Perhaps this patient has a mutation of the deiodinase that converts T4 to T3. There actually are two such enzymes, the types 1 and 2 deiodinases [5]. The type 1 deiodinase is primarily expressed in the liver and is thought to account for a substantial fraction of the circulating T3. The type 2 deiodinase is expressed in the brain, pituitary, brown fat and a few other locations, and is thought to provide local T3 that may or may not contribute substantially to the T3 in the circulation. A major problem invoking the diagnosis of deiodinase mutation is that no such cases have ever been described.

All three deiodinases are selenoproteins, unusual enzymes that contain the amino acid selenocysteine in the catalytic site. The human genome encodes approximately two dozen selenoproteins, and the synthesis of these proteins requires a series a highly specialized enzymes. Several patients have been described who have a mutation in one of those proteins, selenocysteine binding protein 2 (SECIS binding protein 2, SBP2). These patients have a generalized defect in the ability to produce selenoproteins, although not all selenoproteins are equally affected. Subjects with SBP2 mutations have elevated FT4 and low FT3, but the TSH is normal or just slightly elevated, and the thyroid is not enlarged [6]. Thus, our patient does not fit the picture seen with SBP2 mutations. However, only a few cases have been reported, so it is possible that the laboratory findings may expand if more cases are identified.

When a patient is found to have unusual thyroid function tests, and especially if the clinical presentation and test results do not fit well with a known syndrome, it is important to consider the possibility of laboratory artifact. In our patient, the combination of elevated TSH, elevated FT4 and low FT3 should raise this possibility. But which of these tests would be subject to artifact? The elevated TSH seems likely real because it is consistent with two clinical correlates – goiter and an enlarged pituitary. Since the mildly low FT3 is consistent with the elevated TSH, attention turns to the validity of the FT4 measurement. The commonly used clinical FT4 assays are called “analog” methods because a labeled T4 analog competes with the serum free T4 for binding to an antibody. These assays occasionally can give spurious results due to interfering substances in the serum. In fact, although it is generally agreed that FT4 assays are more reliably interpreted than the old-fashioned total T4 assay, some have questioned this [7]. One such possibility is familial dysalbuminemic hyperthyroxinemia. It is caused by mutations in the albumin gene that increases the affinity of albumin for T4 by ~60-fold. Some assays that rely on the competition of a T4 analog with unbound T4 in the sample can give falsely-elevated results. Doing a “two-step” assay, in which T4 analog never comes in contact with mutant albumin due to a wash step before capture avoids this problem. However, the Siemens Centaur assay employed in our laboratory performs as well or even better than some of the two-step assays [8]. Thus, this possibility does not appear to explain the high free T4 values in this patient. Another possibility is the so-called HAMA (human anti-mouse antibody) phenomenon. It is most often encountered in people who are chronically exposed to mouse proteins (home mouse infestation, vivarium or pet store workers etc.). The endogenous heterophile human antibodies against mouse proteins may interfere in the assays based on mouse-derived monoclonal antibodies and give spuriously high results. Therefore, performing an assay utilizing patient’s plasma treated with a blocking reagent is indicated in this case.

**A HAMA assays for FT4 was performed, but the measured concentration of the analyte did not change: 6.2 ng/dL**

In situations where the validity of a FT4 assay is in question, it is sometimes necessary to measure the total hormone.

**In this patient, total T4 was only 3.6 (Normal: 4.5-10.9 mcg/dL) with a normal thyroxine binding globulin of 21 (Normal: 11–27 mcg/mL).**

The total T4 concentration is obviously low and fully compatible with elevated TSH and low T3 concentrations. This strongly suggests that the original suspicion of Hashimoto’s thyroiditis with primary hypothyroidism was actually correct despite apparently elevated FT4 levels measured by the analog method. Therefore, the gold standard of FT4 measurement, i.e. equilibrium dialysis, needs to be performed to clinch the diagnosis.

**Free T4 measured by equilibrium dialysis, was <0.5 (Normal: 0.8-2.0 ng/dL). In addition, the reverse T3 was low at 6.8 (Normal: 10–24 ng/dL), which is the expected result if the true free T4 is low and the patient is hypothyroid.**

## Summary

Thus, the overall results are consistent with a diagnosis of hypothyroidism, with an artifactual elevation in the originally-measured FT4 due to the presence of an unknown interfering substance.

It is noteworthy that this patient has very high titers of thyroglobulin and thyroid peroxidase antibodies, suggesting a diagnosis of Hashimoto’s thyroiditis. Very high titers of thyroglobulin antibodies can be associated with endogenous thyroid hormone antibodies, since T4 (and T3) molecules are present within mature thyroglobulin. These thyroid hormone autoantibodies can be a source of assay interference and can result in spuriously elevated FT4 measurements in the typical clinical assays that use analog methods [9]. Thyroid hormone autoantibodies also would be expected to cause artifacts in total T4 assays, but assays that measure free T4 by equilibrium dialysis are much less susceptible. Although the exact nature of the interfering substance in this patient is not known, the overall laboratory picture leads to the diagnosis of hypothyroidism, and thus informs the clinician of the need to treat with levothyroxine. In addition, it is clear that standard clinical FT4 assays should be avoided in evaluating thyroid function in this patient.

## Consent

Written informed consent was obtained from the patient for publication of this case report and any accompanying images. A copy of the written consent is available for review by the Editor-in-Chief of this journal.
